# Treatment of Diseases of the Mouth

**Published:** 1884-08

**Authors:** Julien W. Russell

**Affiliations:** Brooklyn, N. Y.


### TREATMENT OF DISEASES OF THE MOUTH.

JULIEN W. RUSSELL, M. D. S., BROOKLYN, N. Y.

In the May number of the INDEPENDENT PRACTITIONER there is a query in regard to the use of Homœopathic remedies in the treatment of diseases of the mouth. My experience has proved that remedies may be used in much less strength than they are commonly employed, and produce results that could seldom be attained by the use of heroic doses. For this purpose I use the so-called "Homœopathic remedies," as they are prepared in a certain manner, and one can tell at a glance how much of the original drug is contained in each dilution or trituration. A word here in regard to the preparation of these remedies will not come amiss. "The object is so to prepare each medicinal substance that the whole of its virtues shall be present in a suitable form for administration." The tinctures are prepared either by expressing the juice and mixing it with alcohol, or by maceration in the same fluid: in each case the medicinal substance is contained in the tincture in the proportion of one to two; this is called the "mother tincture.

To prepare the "dilutions" or "potencies," two parts of the mother tincture are added to eight parts of alcohol, which makes the first decimal dilution; to make the second, one part of the first is added to nine parts of alcohol; the third is made by taking one part of the second, and so on; these dilutions are prepared on the "decimal scale."

A great many of the remedies are prepared on the "centesimal scale"; one part of the tincture being added to ninety-nine parts of alcohol, which makes the first centesimal dilution. The second is made from the first in the same manner. The dilutions of the mineral substances when soluble are made with water, as also are the mineral acids.

The metals and their insoluble salts are prepared by the process of trituration. One part of the substance used is rubbed up with either nine or ninety-nine parts of sugar of milk, according as they are prepared on the decimal or centesimal scale; this makes the first trituration. The second is prepared from the first in the same manner. The potencies ordinarily used range from the first to the third. In our specialty I have seldom given higher than the twelfth, and generally use the first, second or third.

In this paper when a potency is mentioned, it will be understood that the centesimal scale is intended. When the decimal is meant it will be so stated. The process of trituration has been of great benefit, as by it many substances have been added to the materia medica, which, before this process was discovered, were unavailable on account of their insolubility.

Gold, Platina, Silver, Tin, and other metals, all valuable remedies, are soluble in the stomach when triturated.

The different salts of calcium are triturated and are specially valuable for treating children, as they are tasteless, and as much benefit can be derived from them as by using the different solutions and syrups.

In cases of different dentition the use of these salts will give good results, and when the child is young it can be mixed with its food without any danger of injury to the stomach. In regard to the repetition of the dose, there seems to be a great diversity of opinion. Some practitioners give it at long and others at short intervals. The general rule is, for acute diseases, to repeat the dose from every fifteen minutes to two hours, and in chronic diseases from every two to six hours. I have found that every two hours is frequent enough except in acute cases of neuralgia or periostitis, when the dose should be repeated every fifteen or thirty minutes, until a change for the better has taken place. The size of the dose is, for triturations, as much as can be taken on the point of a pen-knife, dry on the tongue. If desirable, place in half a glass of water as much of the powder as can be placed on a ten-cent piece; of this one teaspoonful should be taken at a time. When the tinctures are used, put five or ten drops in the same quantity of water, one teaspoonful as a dose.

There are several remedies spoken of in this article which are peculiar to the Homœopathic Pharmacopœia; one of these is the combination of sulphur and calcium, called Hepar sulphuris calcareum. This is prepared by mixing equal portions of powdered oyster shells and pure flowers of sulphur. The mixture is placed in a sealed crucible and kept at a white heat for ten minutes. The resulting salt is known in chemistry as Sulphide of calcium. The potencies are made from this by trituration. There is no space here to describe at length all the instances where it is indicated. I will only say that it seems to have control over the suppurative process, promoting it when desirable, and checking it when excessive.

Mezereum is the spurge-olive; from this the tincture is made by expressing the juice. It has a long-standing reputation as a remedy for periosteal affections.

I have found it very useful as a remedy for periostitis of the jaws; it is also a remedy for rheumatic periostitis, one of the most troublesome diseases that come into our hands for treatment. The last of these remedies that calls for particular notice is the Tarantula cubensis. This is the poison of the Cuban spider. The tincture is prepared by placing a spider in a small bottle of alcohol, when it immediately throws out its poison; from this the dilutions are made. This remedy acts somewhat like Hepar sulph. in healing abscesses. I generally give it in alternation with either Calcarea phos. or Silicea; the sixth decimal dilution is the best.

The chemical names of the remainder of the remedies will sufficiently indicate their composition. Some may say that the average dentist does not possess the requisite knowledge to administer remedies internally, but they must remember that dentistry is becoming more and more each day a specialty of medicine, and those who do not want to be left in the rear should go to work and read up, and while studying principles and practice let them read a few works on Homœopathic Practice, and they will find there many things that will be of assistance to them.

Take, for instance, one of the most common diseases that the dentist is called upon to treat, *i. e.* facial neuralgia.

The patient complains of severe darting pains in one or all of the branches of the fifth nerve; severe toothache on taking anything hot or cold into the mouth. On examining the teeth nothing can be found out of order, no pulps exposed, no signs of periostitis, everything looking healthy; very frequently the pain is located in some tooth that is perfectly sound. Again, the complaint is that every tooth on one side of the jaw is aching; one day it may be in the upper, and the next the lower jaw.

Upon inquiry it will be found that the patient has taken cold in the side of the face by sitting in a draft, or something of the kind. In such cases Aconite is the best remedy. The third centesimal dilution is usually strong enough, though I have cured some cases in a few hours by means of the thirtieth.

When there is marked local hyperæmia, with the face flushing on the affected side at each paroxysm and the eyes are inflamed, Belladonna is to be given. Sometimes alternated with Aconite will have good effect.

When the pain is deep seated, and especially when it is located in the antrum, Arsenicum is to be used. The higher potencies are the best; I generally give the sixth. Another form: When the pain is in the lower jaw and extends down along the side of the neck and there is an absence of fever, Mezereum first will give good results. It would be impossible here to describe all the varieties of this disease and their treatment. There is only room to give some of the remedies: Chamomilla, Phosphorus, Coffea, Kali bromidum, Mercurius, Pulsatilla, and Ignatia. Pregnant women are very frequently sufferers from this disease, which cannot be cured by the ordinary neuralgic remedies. In such cases Sepia, Magnesia, or Calcarea carbonica are required. In scrofulous diseases these attenuations show forth at their best; in abscesses of the jaws, where there is a large quantity of thick, flaky pus secreted with a decided odor, the following method of treatment will usually be successful: Syringe out the pus cavity with a dilute solution of Carbolic acid, and right here your local treatment may stop, for it is rarely necessary to inject more than once. Give the 6th decimal of Tarantula cubensis and the 1st decimal of Calcarea phosphoricum, alternately every hour. In twenty-four hours a decided change will have taken place; the swelling will have decreased, there will be a less quantity of pus secreted, and it will be more healthy in appearance. Apply to the fistula a small piece of lint covered with vasaline, and over this place a bandage to keep it in place. Have the patient change it twice a day. This will serve a twofold purpose, not only keeping the surrounding parts clean and healthy, but a portion of the vasaline enters the fistula, and acts the same as a cotton tent to keep it open.

As a tonic, give Syrupus ferri iodidi, fifteen drops after each meal; also direct cold baths with hand-rubbing afterward. The abscess commences to heal from the inside, and its decrease in size can be measured by means of a probe. In from ten days to two weeks the fistula will have closed, and all that remains will be a small lump, which is slightly painful to the touch. Kali hydriodicum, or Mercurius protiodide will soon cause this to disappear. The above plan I have pursued in a number of cases with decided success. Variations from this method will be necessary, sometimes when complications occur. To hasten the pointing of an abscess, give Hepar sulphuris, or Silicea 3d. In simple abscesses these will often effect a cure without the use of any other remedy. Suppuration of the antrum is commonly met with in scrofulous children. A slight blow on the cheek, or any injury to the teeth will give rise to this disease; its diagnosis is simple; the swelling under the eye varies in size from that of a pigeon's to a hen's egg; yields slightly to pressure and is very sore to the touch. Here, again, are Tarantula and Calcarea phos., the remedies called for. I have treated recently four cases with these remedies, and in all of them the swelling disappeared in from one to three days. Enlargement of the lymphatic and of the submaxillary and sublingual glands are frequently met with in scrofulous subjects. If the case is seen before suppuration has commenced, a cure will result by the use of Sulphur or Calcarea carb. If, on the contrary, suppuration has taken place, they should be treated the same as a simple abscess, by means of Silicea or Hepar sulph. I have devoted some space to the treatment of scrofulous diseases, as I wish to urge the effectiveness of this course of treatment. While writing about children I would like to direct attention to a curious fact in relation to Kreosote. Our use of it has always been locally, to relieve a toothache caused by caries. It may surprise some to know that it has its good effects when given internally. M. Leste recommends it very highly as a specific remedy in difficult dentition. When there is general irritation and the child is fretful and sleepless, it will act very well. He also states that it is a good remedy for toothache when caused by caries, but that I cannot substantiate. I think that it would be successful where there is a difficult eruption of the wisdom tooth. The sixth, twelfth and thirtieth dilutions are to be used. In cases of periostitis I have given the mercurials with good results; Merc. sol., Merc. protiod., and in some cases Merc. corrosivus in the lower potencies from the first to the third, are to be given; Mezereum, Kali bichromicum and Kali bromidum are also effective. When there is much fever Aconite is to be given in alternation with Mezereum. If necrosis has taken place the treatment should be different. Extract the offending teeth and make a number of openings to allow the pus to escape readily. Give Hepar sulph. 1st, or Silicea; if they are slow in acting give Calcarea phos. in alternation with Tarantula. This will effectually check the secretion of pus and prevent the system from being weakened. Tonics are to be given when required. After this, all that can be done is to wait until the sequestrum becomes detached. To assist this process Symphytum has been highly spoken of. In phosphor-necrosis the remedies should be Phosphoricum and Acidum phosphoricum. Caries of the maxilla is the only disease that I have not attempted to cure entirely without the aid of the knife; the remedies for this are Aurum muriaticum, Silicea, Fluoric acid, and in some instances Phosphorus. A large number of cures have been reported by the use of these remedies without surgical interference.

In injuries to the jaws, caused by blows, etc., Arnica is to be given at short intervals. I once had a case of a badly united fracture of the inferior maxilla, where the tongue remained partially paralyzed so that the patient could not speak distinctly. I gave the tincture of Aconite and Belladonna, alternate every hour, and in two days the tongue became perfectly natural. It is impossible for me in the limits of such a paper as this to discuss all the diseases that come into the hands of the dentist, and their appropriate treatment. If by here giving the results of my own experience I shall be able to call the attention of others to the assistance that these remedies will render to the intelligent practitioner, the object of this paper will be achieved.

